# Probiotics in the fight against necrotizing enterocolitis: a cost-effective yet underutilized strategy in neonatal care

**DOI:** 10.1097/MS9.0000000000003944

**Published:** 2025-09-23

**Authors:** Qadr Amir, Gyaneshwari, Maliha Khalid, Muhammad Talha, Aminath Waafira

**Affiliations:** a Department of Medicine, Fatima Jinnah Medical University, Lahore, Pakistan; b Liaquat University of Medical & Health Sciences; c Jinnah Sindh Medical University, Karachi; d King Edward Medical University, Lahore, Pakistan; e Department of Medicine, School of Medicine, The Maldives National University, Malé, Maldives

**Keywords:** necrotizing enterocolitis, neonatal care, preterm infants, probiotics

## Abstract

Necrotizing enterocolitis (NEC) remains a leading cause of gastrointestinal morbidity and mortality among preterm and very low birth weight infants. Probiotics, particularly multi-strain formulations including *Bifidobacteria, Lactobacillus*, and *Enterococcus*, have shown considerable promise in reducing the incidence and severity of NEC by modulating the neonatal gut microbiome, enhancing mucosal barrier integrity, and reducing systemic inflammation. Recent meta-analyses confirm their efficacy in lowering NEC risk and associated mortality. Nevertheless, routine use in clinical practice remains limited due to heterogeneity in neonatal intensive care unit practices, uncertainty around optimal strains, dosage, and treatment duration, and insufficient long-term safety data. Given their cost-effectiveness and strong evidence base, integrating probiotics into standard neonatal care protocols, while addressing existing research gaps, could significantly improve outcomes for this vulnerable population.

*To the Editor,*

Necrotizing enterocolitis (NEC) remains one of the leading causes of gastrointestinal morbidity and mortality among preterm infants, especially those of very low birth weight. The condition – characterized by intestinal inflammation, necrosis, and in severe cases, perforation – affects up to 10% of this vulnerable population. Though multifactorial in origin, recent research has consistently implicated intestinal dysbiosis and aberrant microbial colonization patterns as key drivers in NEC pathogenesis, making the neonatal microbiome a focal point for preventive interventions^[1]^. This article is in line with the TITAN Guidelines on the need for transparency in artificial intelligence use in healthcare^[2]^.

In response, microbiome-guided probiotic supplementation, particularly with multi-strain formulations that include *Bifidobacteria, Lactobacillus*, and *Enterococcus* species, has emerged as a targeted strategy for NEC prevention. A comprehensive umbrella meta-analysis of randomized controlled trials published in 2025 reported that probiotics significantly lowered the risk of NEC (relative risk [RR] ≈ 0.51; 95% CI: 0.46–0.55) and reduced all-cause mortality (RR ≈ 0.72; 95% CI: 0.68–0.76) in preterm infants compared to placebo^[3]^. These encouraging outcomes highlight the potential of personalized, microbiome-directed probiotic regimens to become a staple in NEC prophylaxis.

Emerging studies have shown that probiotics reduce the risk of NEC, neonatal sepsis, and associated mortality rate in preterm infants. According to a 2024 systematic review, administering *Lactobacillus* species alone or a combination of strains significantly decreased the risk of severe stage II to stage III NEC in preterm infants (less than 37 weeks gestation) or infants weighing less than 2500 g at birth^[4]^. This is because *Lactobacillus* and other such probiotic strains can induce the production of mucin, resulting in the proliferation of the mucous layer in the gut^[5]^. Probiotics also strengthen tight junctions between intestinal epithelial cells and reduce exposure to toxins by increasing gastrointestinal motility^[6,7]^.

However, despite the promising outcomes attributed to the use of probiotics in the prevention of NEC, we see that they are not being incorporated into any standardized treatment plan. This could be due to a lack of data on the optimal dose and duration of probiotic therapy^[3]^. In addition, neonatal care techniques, like feeding tactics (breast milk vs. formula milk), antibiotic use, and neonatal intensive care unit protocols, vary around the world, contributing to variation in probiotic efficacy. Furthermore, there is very little data available on the long-term effects of giving probiotics to premature newborns. There is also little research done on the safety profile of giving probiotics to very preterm neonates.

Probiotics have shown promising results in the prevention of NEC and associated mortality in preterm and very preterm infants. Furthermore, it is quite inexpensive, so it does not place an undue demand on resources. Efforts should be made to integrate this therapy into routine medical care. More study is needed to establish a consistent dosage and duration of probiotic therapy, as well as the safety profile associated with its administration. Probiotics, when used properly, can play a vital role in the prevention and treatment of NEC.

## Data Availability

No data were generated for this manuscript.

## Research registration unique identifying number (UIN)

Name of the registry: N/A. Unique Identifying number or registration ID: N/A. Hyperlink to your specific registration (must be publicly accessible and will be checked): N/A.
